# A prognostic and predictive computational pathology immune signature for ductal carcinoma in situ: retrospective results from a cohort within the UK/ANZ DCIS trial

**DOI:** 10.1016/S2589-7500(24)00116-X

**Published:** 2024-07-09

**Authors:** Haojia Li, Arpit Aggarwal, Paula Toro, Pingfu Fu, Sunil S Badve, Jack Cuzick, Anant Madabhushi, Mangesh A Thorat

**Affiliations:** Department of Biomedical Engineering (H Li PhD, P Toro MD) and Department of Population and Quantitative Health Sciences, School of Medicine (P Fu PhD), Case Western Reserve University, Cleveland, OH, USA; Department of Biomedical Engineering, Emory University and Georgia Institute of Technology, Atlanta, GA, USA (A Aggarwal MEng, Prof A Madabhushi PhD); Department of Pathology and Laboratory Medicine, Emory University School of Medicine, Atlanta, GA, USA (Prof S S Badve MD); Winship Cancer Institute, Atlanta, GA, USA (Prof S S Badve); Centre for Cancer Screening, Prevention and Early Diagnosis, Wolfson Institute of Population Health, Queen Mary University of London, London, UK (Prof J Cuzick PhD, M A Thorat PhD); Joseph Maxwell Cleland Atlanta VA Medical Center, Atlanta, GA, USA (Prof A Madabhushi); School of Cancer & Pharmaceutical Sciences, Faculty of Life Sciences & Medicine, King’s College London, London, UK (M A Thorat); Breast Surgery, Homerton University Hospital, London, UK (M A Thorat); Breast Surgery, Guy’s Hospital, Great Maze Pond, London, UK (M A Thorat)

## Abstract

**Background:**

The density of tumour-infiltrating lymphocytes (TILs) could be prognostic in ductal carcinoma in situ (DCIS). However, manual TIL quantification is time-consuming and suffers from interobserver and intraobserver variability. In this study, we developed a TIL-based computational pathology biomarker and evaluated its association with the risk of recurrence and benefit of adjuvant treatment in a clinical trial cohort.

**Methods:**

In this retrospective cohort study, a computational pathology pipeline was developed to generate a TIL-based biomarker (CPath TIL categories). Subsequently, the signature underwent a masked independent validation on H&E-stained whole-section images of 755 patients with DCIS from the UK/ANZ DCIS randomised controlled trial. Specifically, continuous biomarker CPath TIL score was calculated as the average TIL density in the DCIS microenvironment and dichotomised into binary biomarker CPath TIL categories (CPath TIL-high *vs* CPath TIL-low) using the median value as a cutoff. The primary outcome was ipsilateral breast event (IBE; either recurrence of DCIS [DCIS-IBE] or invasive progression [I-IBE]). The Cox proportional hazards model was used to estimate the hazard ratio (HR).

**Findings:**

CPath TIL-score was evaluable in 718 (95%) of 755 patients (151 IBEs). Patients with CPath TIL-high DCIS had a greater risk of IBE than those with CPath TIL-low DCIS (HR 2·10 [95% CI 1·39–3·18]; p=0·0004). The risk of I-IBE was greater in patients with CPath TIL-high DCIS than those with CPath TIL-low DCIS (3·09 [1·56–6·14]; p=0·0013), and the risk of DCIS-IBE was non-significantly higher in those with CPath TIL-high DCIS (1·61 [0·95–2·72]; p=0·077). A significant interaction (p_interaction_=0·025) between CPath TIL categories and radiotherapy was observed with a greater magnitude of radiotherapy benefit in preventing IBE in CPath TIL-high DCIS (0·32 [0·19–0·54]) than CPath TIL-low DCIS (0·40 [0·20–0·81]).

**Interpretation:**

High TIL density is associated with higher recurrence risk—particularly of invasive recurrence—and greater radiotherapy benefit in patients with DCIS. Our TIL-based computational pathology signature has a prognostic and predictive role in DCIS.

**Funding:**

National Cancer Institute under award number U01CA269181, Cancer Research UK (C569/A12061; C569/A16891), and the Breast Cancer Research Foundation, New York (NY, USA).

## Introduction

Ductal carcinoma in situ (DCIS) is stage 0 breast cancer with the morphologically and biologically diverse carcinoma cells confined within the breast duct. DCIS accounts for approximately 15–20% of newly diagnosed breast cancer cases.^1^ Adjuvant radiotherapy reduces by half the ipsilateral breast event (IBE) rate in patients with DCIS treated by breast-conserving surgery.^2^ However, this reduction in IBE does not translate in improving overall survival.^3^ Additionally, for the patients with low-risk DCIS, the radiotherapy benefit might not outweigh the harms (including financial burden) of radiotherapy.^4^ Currently, the use of adjuvant radiotherapy is mainly guided by pathological tumour size and nuclear grade.^3^ However, the specific guidelines vary widely among different regions of the world and often lead to overtreatment in clinical practice.^5^ Moreover, the nuclear grade assigned by pathologists from visual inspection is often subject to interobserver or intraobserver variability with low reproducibility.^6^ Although a multitude of molecular assays have been developed for the risk stratification of DCIS,^7,8^ these assays have not been adequately and widely validated and no robust molecular biomarkers are routinely used in clinical practice yet. Therefore, objective prognostic and predictive biomarkers to accurately risk-stratify patients are urgently needed.

The role of tumour-infiltrating lymphocytes (TILs) as prognostic and predictive markers in invasive breast cancer has been well established.^9^ However, although studies exploring the clinical significance of density, distribution, or composition of TILs in DCIS are gaining popularity, the associated data remain scarce compared with invasive breast cancer. A small number of studies have shown the prognostic values of the density of TILs present in the DCIS microenvironment.^10–16^ However, some of these studies^10–13^ are limited by relatively small sample size or potential treatment-related confounding. In addition, all but one^16^ of the studies counted the lymphocytes manually. Although previous work has shown a high reproducibility in manual assessment of TILs in DCIS under pre-planned detailed criteria,^17^ it is still a relatively time-consuming task in clinical practice and requires a substantial level of expertise.

With the advent of digital pathology, there has been substantial interest in developing and applying computational tools to quantitatively explore and measure diagnosis-related and prognosis-related tumour morphology characteristics. The use of machine learning in digital pathology enables an objective and reproducible measurement of tumour morphology and is now readily feasible with the progressive digitalisation of pathology laboratories. Therefore, it could potentially provide assistive tools for pathologists in clinical practice by generating quantitative metrics working in tandem with the manual visual examination. We present a computational pathology pipeline evaluating the microenvironment of manually annotated DCIS regions to categorise patients into two different TIL categories (CPath TIL-high: high density of TIL in DCIS microenvironment; CPath TIL-low: low density of TIL in DCIS microenvironment) and blinded (ie, TIL scores were generated without outcome data being linked) evaluation of the prognostic and predictive value of this biomarker in the UK, Australia, and New Zealand (UK/ANZ) DCIS trial.^2^

## Methods

### Study design

This retrospective cohort study was conducted in all UK/ANZ DCIS participants for whom pathology material of tumour tissue was available. Formal power calculations were not performed for this retrospective study; CPath TIL categories were validated in all available samples. The UK/ANZ clinical trial was conducted in the UK, Australia, and New Zealand between May, 1990, and August, 1998 to investigate the effect of tamoxifen and radiotherapy for patients with locally excised DCIS. The samples used in this study came from 36 hospitals in the UK.

A nested case–control design using matching by treatment allocation was used in sensitivity analyses of the prognostic role of CPath TIL categories to minimise residual treatment-related confounding. Biomarker measurement was carried out by the first author masked to the study outcome and clinicopathological variables. This study is reported in accordance with the REMARK criteria.^18^ The collection of pathology material and its use in biomarker studies was approved by the National Research Ethics Service—Joint UCL/UCLH Committees on Ethics of Human Research (Committee Alpha).

### Study population

The UK/ANZ DCIS trial^2^ is a randomised 2 × 2 factorial trial of 1694 eligible patients with DCIS who were randomly assigned into one of the four adjuvant treatment groups (no adjuvant treatment, radiotherapy alone, tamoxifen alone, and both radiotherapy and tamoxifen) following a complete wide local excision. After a median follow-up of 12·7 years (IQR 10·9–14·7), there have been 162 invasive breast cancer and 197 DCIS events (17 unknown, 376 total). The patient cohort (n=755) included in this study—biomarker study subset 1 (BSS1; appendix p 2)—has been described previously.^19,20^ BSS1 comprises formalin-fixed paraffin embedded tissue blocks of DCIS collected from 36 hospitals in the UK. BSS1 was similar to the remaining trial population with regard to treatment allocation and other clinicopathological factors but contained a significantly higher proportion of high cytonuclear grade DCIS^21^ and DCIS with necrosis and larger lesions (appendix p 4). Patients with DCIS who had micro-invasion were excluded from BSS1. The H&E-stained whole sections from formalin-fixed paraffin-embedded blocks were digitised into whole-section images (WSIs) using the 3DHistech P250 scanner (3DHistech, Budapest, Hungary) at 43× magnification.

### Procedures

For each patient in BSS1, we calculated CPath TIL categories in DCIS-associated microenvironments. The workflow for the extraction of computational pathology signature (ie, CPath TIL categories) is displayed and described in the appendix (pp 9–14). Specifically, the DCIS-bearing area was manually annotated by a breast pathologist, except the areas deemed too small for analysis. The microenvironment for a DCIS was defined as the periductal region within a radius of 250 μm of the DCIS boundary. An approach combining a pretrained nuclei segmentation model^22^ and image-processing technique was developed to segment the stroma in the microenvironment as described in the appendix (pp 10–11, 14). Subsequently, the stroma in the DCIS microenvironment was partitioned into small image patches. The percentage of TILs present in each patch was predicted by a pretrained convolutional neural network.^23^ A semi-quantitative manual validation was performed by an experienced board-certified breast pathologist to show the validity of the pre-trained convolutional neural network model for TIL classification on BSS1 (appendix p 12).

The raw CPath TIL score was multiplied by ten for ease of interpretation of data distribution and effect sizes. CPath TIL score was further dichotomised into CPath TIL-low (score less than threshold) and CPath TIL-high (score equal to or greater than threshold) groups by applying the pre-defined median score as the threshold. CPath score is a continuous variable and CPath category is a binary variable created by applying a threshold on CPath score.

A biomarker reproducibility analysis was implemented as described in the appendix (p 15). In addition, to investigate the robustness of the CPath TIL score in relation to the selection of the microenvironment radius, a radius of 200 μm was used to generate the CPath TIL score.

### Statistical analysis

Full details on the trial procedures, follow-up,^2^ and histopathology review^24^ have been reported previously. The histopathology review was performed by an experienced breast pathologist to assess several important histopathological features on slides of representative tumour tissues from the surgical excision. The variables assessed included excision margins and completeness of excision, tumour size, cytonuclear grade, necrosis, and periductal inflammation. The primary outcome used in this study is IBE (either recurrence of DCIS [DCIS-IBE] or invasive progression [I-IBE]). Only the first new IBE was considered. Missing data were not imputed. The patients who did not have an IBE until the date of last follow-up (Oct 1, 2008) were censored.

All p values are two-sided and a p value of 0·05 or less was deemed significant. Incremental improvement of models was based on differences in χ^2^ values from respective likelihood ratio tests. Statistical analyses were performed using Stata (version 16.0) and Review Manager (version 5.3; The Nordic Cochrane Centre, Copenhagen, Denmark).

For the time-to-recurrence analyses, the Cox proportional hazard model was used to estimate the hazard ratio (HR). 10-year estimates and survival plots were produced by the Kaplan–Meier method. IBE was the primary outcome, and additional analyses with DCIS-IBE and I-IBE as secondary outcomes were also performed.

In addition, we performed subgroup analyses to assess clinical utility of CPath TIL categories in patients with completely excised low-grade or intermediate-grade DCIS, for whom there is considerable debate regarding adjuvant treatment. Furthermore, the Kaplan–Meier method was used to estimate 10-year IBE, I-IBE, and DCIS-IBE rates by the receipt of radiotherapy in subgroups with CPath TIL-high and CPath TIL-low DCIS.

Sensitivity analyses of the prognostic role of CPath TIL categories restricted to the nested case–control study were undertaken to minimise any residual treatment-related confounding. Patients who developed IBE during the follow-up (ie, up until the last follow-up date) were matched to those who did not develop IBE during follow-up (ie, the control group) by age and treatment allocation using a 1:2 case–control ratio (181 patients and 362 controls), and controls had to be followed up for at least as long as the time to event in their matching case (appendix p 3). The participants in the control group could be up to 7 years younger or 7 years older than the participant in the case group, at the time of entry in the trial. In the nested case–control study, analyses of the risk of recurrence by CPath TIL categories were performed by a conditional logistic regression model (IBE as primary case-definition) to estimate matched odds ratios (ORs).

### Role of the funding source

Funders had no role in study design, data collection, data analysis, data interpretation, or writing of the report.

## Results

CPath TIL score could be quantified in 718 (95%) of 755 patients after excluding WSIs without DCIS or too large to be computationally processed. TILs data acquisition period was from Nov 1, 2020, until Dec 16, 2021. CPath TIL score (raw density multiplied by ten) ranged from 0·04 to 4·97, with a mean score of 0·90 (SD 0·64) and median score of 0·71 (IQR 0·50–1·08). The reproducibility analyses showed that the Spearman correlation coefficient between the original and remeasured (by a different segmentation algorithm, see appendix p 15) CPath TIL score and CPath TIL categories was 0·95 and 0·98, respectively. The Spearman correlation coefficient for the CPath TIL score and CPath TIL categories between a radius of 200 μm and a radius of 250 μm was 0·95 and 0·88, respectively. CPath TIL categories remained the same for 675 (94%) of 718 patients. CPath TIL-high score (table 1) was associated with larger lesion size (p<0·0001), higher cytonuclear grade (p=0·0001),^21^ presence of necrosis (p=0·0001), HER2 overexpression (p=0·0001), and lack of oestrogen receptor (ER) expression (p=0·0001; table 1). CPath TIL categories showed good correlation (Spearman rank correlation coefficient=0·58) with periductal inflammation manually estimated^25^ by an experienced breast pathologist (p<0·0001).

In the univariate analyses, CPath TIL-high DCIS had a significantly greater (HR 2·14 [95% CI 1·53–3·00]) IBE risk compared with CPath TIL-low DCIS; I-IBE and DCIS-IBE risks were significantly elevated for CPath TIL-high DCIS; and Kaplan–Meier plots by CPath TIL categories are displayed in the appendix (p 16; table 2). Analyses using CPath TIL-score as a continuous variable showed similar results with significantly elevated IBE and I-IBE risk for CPath TIL-high DCIS (table 2). In 625 (83%) of 755 patients for whom data for both variables were available, CPath TIL categories (HR 2·52 [95% CI 1·76–3·63]) were significantly more (δχ^2^ [one degree of freedom (df)] 18·67; p<0·0001) informative as a prognostic variable than manually estimated^24^ periductal inflammation (HR 2·01 [95% CI 1·21–3·35]).

Sensitivity analyses in the nested case–control study (n=408) showed similar results with greater IBE (matched OR 2·60 [95% CI 1·66–4·09]; p<0·0001) in CPath TIL-high DCIS than in CPath TIL-low DCIS (appendix p 5).

In multivariate analyses, inclusion of CPath TIL categories in the model of clinicopathological and treatment variables significantly improved prediction of recurrence (δχ^2^ [one df] 9·24; p=0·0024) and the HR of CPath TIL categories (HR 2·10 [95% CI 1·39–3·18]) changed very little from its univariate value (2·14 [1·53–3·00]), further supporting its role as an independent predictor of recurrence (table 3).

Data on ER expression are available only in the nested case–control study subset. Therefore, the multivariate analyses in the entire BSS1 excluded ER as a variable. However, the sensitivity analyses in the nested case–control study included ER as a variable (appendix p 6). The sensitivity analyses showed only (as opposed to other variables) CPath TIL categories (matched OR 3·06 [95% CI 1·56–6·03]; p=0·0012) and ER (2·00 [1·04–3·83]; p=0·037) to be independent predictors of IBE.

For interaction analyses, there was no interaction between CPath TIL categories and tamoxifen benefit (p=0·90). The tamoxifen effect did not vary between CPath TIL-high DCIS (HR 0·67 [95% CI 0·45–0·99]) and CPath TIL-low DCIS (0·61 [0·35–1·07]). The interaction term between CPath TIL categories and radiotherapy was significant (p_interaction_=0·025). The magnitude of radiotherapy effect in preventing IBE was greater in CPath TIL-high DCIS (HR 0·32 [95% CI 0·19–0·54]) than in CPath TIL-low DCIS (0·40 [0·20–0·81]), and the magnitude of radiotherapy effect in preventing DCIS-IBE (p_interaction_=0·026) was also greater in CPath TIL-high DCIS (0·27 [0·13–0·55]) than in CPath TIL-low DCIS (0·45 [0·20–1·04]; table 4; figure). The effect of radiotherapy in preventing I-IBE did not differ by CPath TIL categories (p_interaction_=0·50).

Absolute radiotherapy benefit in reducing 10-year IBE rates was more than 20% in CPath TIL-high DCIS (14·3% *vs* 36·1%), and less than 10% in CPath TIL-low DCIS (7·9% *vs* 17·4%; appendix p 8). 10-year I-IBE rate in CPath TIL-low DCIS was 7% even in patients allocated to no adjuvant radiotherapy. Among the 100 patients in BSS1 with completely excised low-grade or intermediate-grade DCIS, 66 (66%) were allocated to no radiotherapy whereas 34 (34%) were allocated to receive radiotherapy. In those allocated to no radiotherapy, four (8%) of 51 patients with CPath TIL-low DCIS and three (20%) of 15 patients with CPath TIL-high DCIS had IBE (appendix p 17). The I-IBE rate in patients with completely excised low-grade or intermediate-grade CPath TIL-low DCIS was 3·9% (95% CI 1·1–16·3) without adjuvant radiotherapy at a median follow-up of 12·7 years (IQR 10·9–14·7).

## Discussion

To the best of our knowledge, this is the first study to show the prognostic and predictive value of computer-extracted TIL density in a randomised controlled trial (RCT) dataset with 718 patients. Although TIL density is not routinely assessed in clinical settings to directly influence treatment selection, it is associated with adverse histopathological features such as high nuclear grade and large lesion size in DCIS^10–16^ factors which determine the use of adjuvant radiotherapy.^7^ Therefore, a treatment bias towards TIL-high DCIS exists, resulting in treatment-related confounding^26^ in observational studies^10–13,15,16^ where treatment allocation was not random. Minimisation of treatment-related confounding factors by assessing TILs in a cohort in which treatment allocation was random is a major strength of our study. Any residual treatment-related confounding was further minimised by performing sensitivity analyses in the nested case–control study. Our proposed computational pipeline circumvents the laborious manual estimation used in previous DCIS TIL studies.^10–15^ Our computational pipeline represents the other two strengths of our study. First, our method is quantitative and reproducible. Second, our biomarker, which directly measures TIL density in the DCIS microenvironment, is highly interpretable and easily appreciated by the clinicians, as opposed to blackbox-based deep learning models. Furthermore, even though a strong positive correlation existed between the CPath TIL categories and manually assessed periductal inflammation, we observed that CPath TIL categories were significantly more informative as a prognostic variable than manually assessed periductal inflammation. The result suggests that our method could quantitatively capture aspects of morphology that might be challenging or too tedious for pathologists to capture visually. In practice, the provided quantitative information regarding the tumour microenvironment could complement the morphological assessment by a pathologist.

Similar to previous studies,^10–16^ we observed that higher TIL density (CPath TIL-high) was associated with larger lesion size, higher cytonuclear grade, presence of necrosis, lack of ER expression, and HER2 over-expression, all of which are adverse prognostic indicators in DCIS.^19,20,24^ However, CPath TIL categories remained independently prognostic in multivariate analyses. CPath TIL-high DCIS had a higher risk of IBE than CPath TIL-low DCIS, and in particular, the risk of I-IBE was three times higher for CPath TIL-high DCIS than for CPath TIL-low DCIS. To the best of our knowledge, the effect size of I-IBE risk observed in this study is the largest among good-quality biomarker studies evaluating I-IBE risk predictors. Our findings are congruent with a number of previous studies showing an association between higher TIL density and higher chance of recurrence and progression of DCIS.^10–15^ For example, Miligy and colleagues^11^ reported a significant positive correlation between the number of stromal B lymphocytes and shorter recurrence-free interval in patients with DCIS. However, most of these previous studies^10–15^ rely on manual counting of TIL in DCIS. Although Hagos and colleagues’ work^16^ computationally quantified the TIL, and they report lower TIL density to be associated with recurrence risk, this study uses a non-representative patient cohort and appears to have substantial overfitting, calling into question the reliability of the results. To our knowledge, our study is the first to show prognostic and predictive role of the computational TIL-based signature in a cohort from an RCT.

In a previous study,^25^ *TP53*, a gene regulating cell proliferation, was found to be more frequently mutated in DCIS with a higher number of TILs. In addition, the TIL number in DCIS was found to be positively correlated with the fraction of the genome altered by copy number.^25^ These identified links between underlying genomic changes and TIL density could provide mechanistic insight into TIL’s prognostic role. Immunoediting could be a contributory mechanism facilitating the gain of mutations that underpin the progression to an invasive disease stage. The specific hypothesis is that in a high TIL microenvironment, DCIS could reach a state of functional dormancy in its silo while simultaneously undergoing immunoediting and gain of mutations. The lesion progresses to the next stage in which the immune response can no longer block tumour outgrowth eventual outcome is tumour cell migration and metastasis.^27^ This hypothesis provides the most plausible explanation behind a greater increase in I-IBE than DCIS-IBE in CPath TIL-high DCIS. Contrary to our study and other studies, the study by Risom and colleagues^28^ suggests a lower stromal immune response in patients with DCIS who have progressed to the invasive disease stage compared with those who have not progressed to the invasive disease stage.^28^ This suggestion is, however, not based on direct quantitative measurements but inferred from gene-set enrichment analysis. Furthermore, the comparisons between patients who have and those who have not progressed to the invasive disease stage in this small study appear to be unreliable because there is probably a strong selection bias and confounding. For example, not only did the two groups differ in the use of adjuvant radiotherapy, but 13 (30%) of 44 patients in the subgroup who did not progress to the invasive disease stage had undergone mastectomy and therefore could not have progressed even if they were biologically predisposed to disease progression.

We observed a significant interaction between CPath TIL categories and radiotherapy. The interaction was not significant for radiotherapy efficacy in preventing invasive events. Absolute radiotherapy benefit in reducing 10-year ipsilateral recurrence rates was more than 20% in CPath TIL-high DCIS and less than 10% in CPath TIL-low DCIS. However, in the only other study—to our knowedge—based on an RCT, Schiza and colleagues^14^ did not observe a significant interaction between TIL density and radiotherapy effect. It is possible that the use of a specific cutoff (≤5% *vs* >5%) that classified 440 (62%) of 711 patients as having TIL-low DCIS could be responsible for the difference in their findings as compared with ours. The other differences in the study populations (eg, higher proportion of high-grade DCIS in the UK/ANZ DCIS trial than in the study by Schiza and colleagues) could also be responsible. A small study by Xu and colleagues^13^ showed that DCIS with sparse touching (≤5 per duct) TILs derived significantly higher benefit of RT than dense touching TILs. The findings from this study are however not robust due to small sample size, small number of events, and proportionately much higher early censoring in the group of patients receiving radiotherapy. We did not observe a significant interaction between adjuvant tamoxifen and CPath TIL categories.

We have previously discussed^7,8^ the importance of new prognostic and predictive markers in DCIS to avoid overtreatment and undertreatment. In the E5194 trial,^29^ the 12-year IBE rate without radiotherapy was found to be 14·4% for the patients with low-grade or intermediate-grade DCIS measuring up to 25 mm. This cohort includes a substantial proportion of patients (with tumour size >10 mm) who would receive radiotherapy as per the European Society for Medical Oncology guidelines.^5^ The IBE rate of 14·4% is neither high nor low, and therefore results in difficulties with adjuvant treatment decisions. 107 (75%) of 142 patients with low-grade or intermediate-grade DCIS in our study were CPath TIL-low. Among patients with completely excised low-grade or intermediate-grade allocated to no adjuvant radiotherapy, only four (8%) of 51 patients with CPath TIL-low DCIS had an IBE at 12·7 years (IQR 10·9–14·7) median follow-up, compared with three (20%) of 15 patients in CPath TIL-high DCIS (appendix p 17). The I-IBE rate was 3·9% (95% CI 1·1–16·3) in patients with CPath TIL-low DCIS. With a high event rate and a greater radiotherapy benefit, patients with low-grade or intermediate-grade CPath TIL-high DCIS would merit adjuvant radiotherapy, whereas those with CPath TIL-low DCIS could safely avoid radiotherapy.

Our study has its limitations. We focused on the quantification of all TILs in the DCIS microenvironment and did not investigate spatial location and composition of TILs. Although these investigations are outside the scope of this study, future work will entail developing a computational approach to account for spatial information (eg, whether the TIL is in direct contact to DCIS basement membrane) and TIL subtypes. Even though this computational pathology pipeline is largely automated, manual annotation of the DCIS regions still needed to be performed as the first step. Future work will entail accurately segmenting the DCIS using a computational model,^16^ obviating the need for manual annotation. Manual assessment of TILs in our study predates the TILs working group guidelines. The TILs working group guidelines would now be considered as a gold standard and the absence of this comparator is a limitation. In addition, the proportion of high-grade tumours (68%) in the UK/ANZ trial is somewhat higher than contemporary datasets. Furthermore, this biomarker study subset comprised marginally larger tumours and had a greater proportion of high-grade DCIS than the remaining trial participants. Such selection bias could have resulted in higher IBE rates than those observed in contemporary datasets. However, underpinning biological effects and observed effect sizes are not likely to have been distorted by selection bias. The absence of ER data in the whole cohort and therefore the absence of ER in the multivariate analyses is a limitation. However, the sensitivity analyses in the nested case–control study including ER in multivariate analyses do not show materially different results, thus supporting the robustness of the prognostic role of CPath TIL categories. Inflammation could be triggered by obesity, which in turn affects outcome and treatment response of patients with breast cancer.^30^ Absence of a BMI variable for correlation analysis with TIL is another limitation of this study. We used a pretrained method for CPath TIL assessment and a median as a pre-defined cutoff in this validation study. However, clinical implementation requires a specific measurement as a cutoff, which will need a prospective study to assess clinical utility. Formal power calculations were not performed for this retrospective study and the observation of the predictive role of CPath TIL for radiotherapy benefit is hypothesis-generating, meriting prospective evaluation. The prospective evaluation of predictive characteristics and clinical utility of this biomarker could be assessed in an RCT of radiotherapy versus no radiotherapy in patients with low-grade or intermediate-grade DCIS with CPath TIL as a stratification variable.

To summarise, we present a computational TIL signature named CPath TIL categories and show that it is prognostic in DCIS, particularly in relation to the risk of invasive recurrence and the prediction of radiotherapy benefit. Our findings highlight the important role of machine-derived TIL quantification in DCIS and provide an automated assistive tool for personalising management in patients with DCIS.

## Supplementary Material

Supplement

## Figures and Tables

**Figure:**
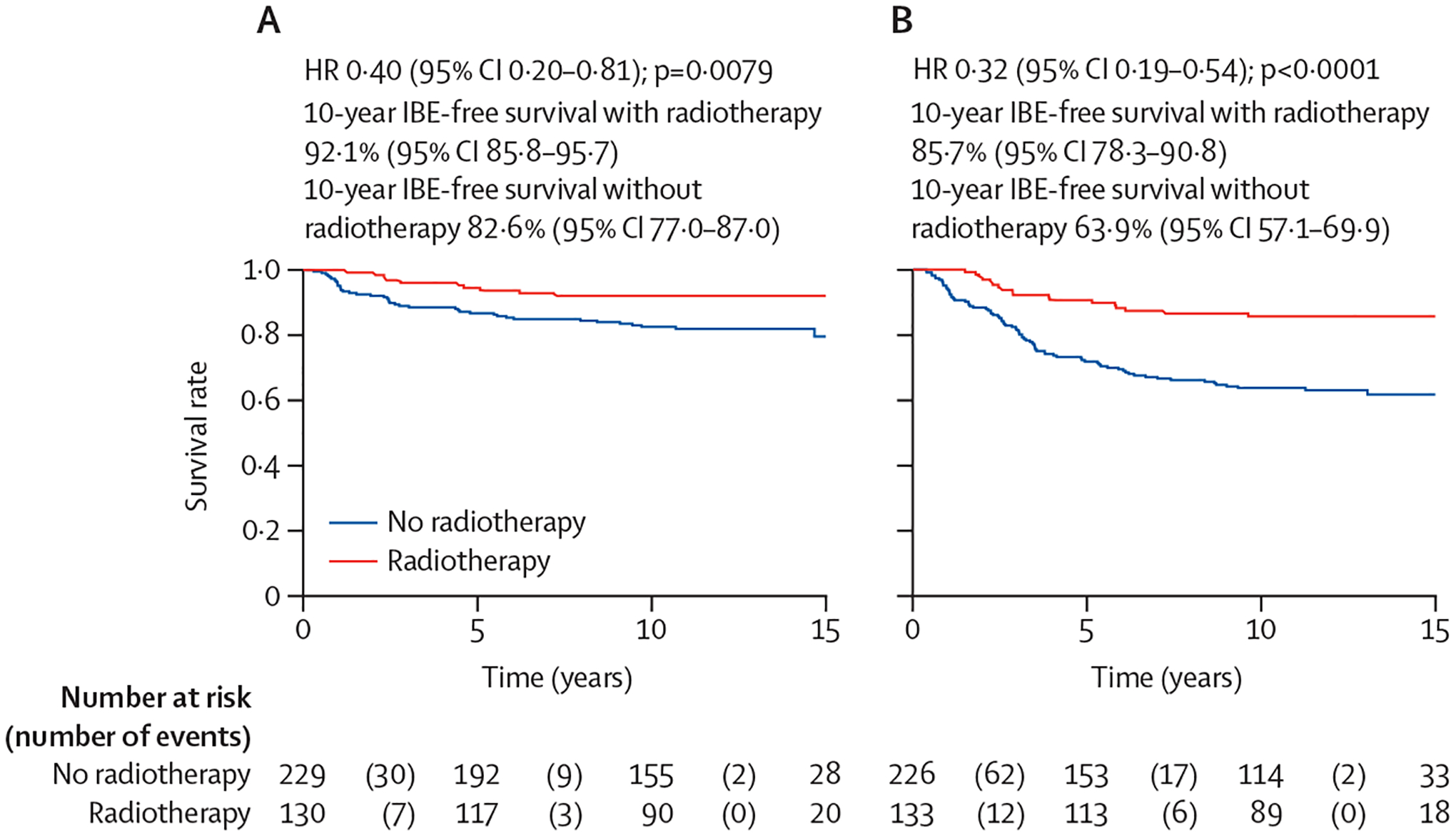
Benefit of radiotherapy in preventing IBE by CPath TIL categories Kaplan–Meier survival plots in (A) Cpath TIL-low DCIS and (B) CPath TIL-high DCIS. DCIS=ductal carcinoma in situ. HR=hazard ratio. IBE=ipsilateral breast event. TIL=tumour-infiltrating lymphocyte. p values are from log-rank tests.

**Table 1:** Distribution of CPath TIL categories by tumour characteristics and age

	Number of patients	CPath TIL-low	CPath TIL-high	p value
Median age (IQR), years	718	56·8 (52·6–61·4)	57·3 (53·7–61·7)	0·30*
Median tumour size (IQR), mm	645	13 (8·5–18·0)	15 (10·0–20·0)	<0·0001*
Grade	641			0·0001†
Low cytonuclear		29/641 (9%)	7/641 (2%)	
Intermediate cytonuclear		78/641 (25%)	28/641 (9%)	
High cytonuclear		208/641 (66%)	291/641 (89%)	
Necrosis	624			0·0001†
Absent		30/624 (10%)	10/624 (3%)	
<10%		47/624 (15%)	25/624 (8%)	
10–50%		83/624 (27%)	65/624 (21%)	
>50%		149/624 (48%)	215/624 (68%)	
ER	485			0·0001†
Negative		47/485 (20%)	135/485 (54%)	
Positive		186/485 (80%)	117/485 (46%)	
*HER2*	689			0·0001†
Negative		270/689 (79%)	182/689 (52%)	
Positive		71/689 (21%)	166/689 (48%)	

Data are n/N (%), unless otherwise stated. p values are comparing differences between patients with CPath TIL-low DCIS and those with CPath TIL-high DCIS. DCIS=ductal carcinoma in situ. ER=oestrogen receptor. TIL=tumour-infiltrating lymphocyte.

* Mann–Whitney test.

† Kruskal–Wallis test.

**Table 2:** Computerised TIL density as a predictor of recurrence, by type of recurrence

	Number of events		CPath TIL score		CPath TIL-category (low vs high)	
	Univariate	Multivariate	Univariate HR (95% CI); p value	Multivariate HR (95% CI); p value	Univariate HR (95% CI); p value	Multivariate HR (95% CI); p value
IBE	151	128	1·37 (1·14–1·66); 0·0011	1·10 (0·87–1·39); 0·43	2·14 (1·53–3·00); <0·0001	2·10 (1·39–3·18); 0·0004
I-IBE	57	47	1·50 (1·12–2·01); 0·0061	1·35 (0·94–1·94); 0·11	2·42 (1·38–4·23); 0·0019	3·09 (1·56–6·14); 0·0013
DCIS-IBE	92	79	1·26 (0·97–1·64); 0·081	0·94 (0·69–1·29); 0·71	1·92 (1·26–2·95); 0·0026	1·61 (0·95–2·72); 0·077

Recurrence type not known in two events in univariate analyses. Multivariate analyses are in a smaller number of samples (n=599) due to non-availability of clinicopathological data in some samples. Multivariate model covariates include age, *HER2* status, completeness of excision, tumour size (mm), cytonuclear grade, necrosis, adjuvant tamoxifen, and adjuvant radiotherapy. p values shown are for each HR (ie, the probability that the HR is purely due to chance). DCIS=ductal carcinoma in situ. DCIS-IBE=ipsilateral recurrence of DCIS. HR=hazard ratio. IBE=ipsilateral breast event. I-IBE=ipsilateral invasive progression. TIL=tumour-infiltrating lymphocyte.

**Table 3:** Multivariate analysis of CPath TIL categories, clinicopathological variables, and treatment variables with IBE as outcome

	Number of patients	Univariate HR (95% CI); p value	Multivariate HR (95% CI); p value
**CPath TIL category**			
Low	290	1 (ref)	1 (ref)
High	309	2·60 (1·77–3·81); <0·0001	2·10 (1·39–3·18); 0·0004
***HER2* status**			
Negative	389	1 (ref)	1 (ref)
Positive (IHC3+)	210	1·97 (1·39–2·79); 0·0001	1·56 (1·07–2·27); 0·0219
**Age**			
Mean (SD), years	57·4 (6·0)	0·98 (0·95–1·01); 0·29	0·98 (0·95–1·01); 0·17
**Excision**			
Complete	406	1 (ref)	1 (ref)
Uncertain	98	1·39 (0·88–2·18); 0·15	1·42 (0·90–2·24); 0·13
Incomplete	95	1·66 (1·07–2·58); 0·023	1·82 (1·16–2·85); 0·0086
Trend test*	NA	NA; 0·015	NA; 0·0058
**Tumour size**			
Mean (SD), mm	15·6 (8·5)	1·03 (1·02–1·05); 0·0001	1·02 (1·00–1·04); 0·027
**Cytonuclear** grade			
Low	34	1 (ref)	1 (ref)
Intermediate	98	1·21 (0·40–3·67); 0·74	0·79 (0·24–2·58); 0·69
High	467	2·15 (0·79–5·82); 0·13	0·95 (0·30–3·02); 0·94
Trend test*	NA	NA; 0·018	NA; 0·7
**Necrosis**			
No	39	1 (ref)	1 (ref)
Yes	560	1·90 (0·78–4·65); 0·16	1·26 (0·45–3·51); 0·66
**Tamoxifen**			
No	278	1 (ref)	1 (ref)
Yes	321	0·64 (0·45–0·91); 0·012	0·69 (0·48–0·98); 0·038
**Radiotherapy**			
No	376	1 (ref)	1 (ref)
Yes	223	0·33 (0·21–0·52); <0·0001	0·31 (0·20–0·49); <0·0001

The outcome is the occurrence of an IBE. 128 (21%) of 599 participants in this analysis had an IBE. Univariate analyses were restricted to the same sample size as available for multivariate analyses. HER2 positive defined as IHC3+ score as per ASCO-CAP 2013 recommendations. p values shown are for each HR (ie, the probability that the HR is purely due to chance). HR=hazard ratio. NA=not applicable. IBE=ipsilateral breast event. IHC=immunohistochemistry. TIL=tumour-infiltrating lymphocyte.

* The trend test evaluates if there is a significant trend across the ordinal subgroups (ie, completeness of excision and grade), from low to high.

**Table 4:** Effect of radiotherapy by Cpath TIL-categories and primary (IBE) and secondary (I-IBE and DCIS-IBE) outcomes

	Number of events	HR (95% CI)	p value	p_interaction_
**IBE**				
CPath TIL-low	51	0·40 (0·20–0·81)	0·010	··
CPath TIL-high	100	0·32 (0·19–0·54)	<0·0001	0·025
**I-IBE**				
CPath TIL-low	18	0·33 (0·09–1·13)	0·077	··
CPath TIL-high	39	0·43 (0·21–0·91)	0·028	0·50
**DCIS-IBE**				
CPath TIL-low	33	0·45 (0·20–1·04)	0·061	··
CPath TIL-high	59	0·27 (0·13–0·55)	0·0003	0·026

Data are for 359 patients. Recurrence type not known in two events in the CPath TIL-high subgroup. DCIS=ductal carcinoma in situ. Interaction p values are for that endpoint, not for each subgroup. DCIS-IBE=ipsilateral recurrence of DCIS. HR=hazard ratio. IBE=ipsilateral breast event. I-IBE=ipsilateral invasive progression. TIL=tumour-infiltrating lymphocyte.

## Data Availability

The code developed and utilised in this study is available from the corresponding authors on reasonable request.

## Research in context

### Evidence before this study

Patients with ductal carcinoma in situ (DCIS) comprise 15–20% of patients with breast cancer in countries with population-based screening for breast cancer. Overtreatment in these patients is a well recognised problem, underscoring the need for biomarkers that will allow personalised treatment. Tumour-infiltrating lymphocytes (TILs) are being increasingly recognised as a prognostic and predictive biomarker in invasive breast cancer. However, the role of TILs in DCIS is still unclear. Only a small number of studies have investigated the role of stromal TILs in DCIS, and these suggest an association between a high density of TILs present in DCIS microenvironment with a poor prognosis. However, some of these studies have relatively small sample sizes and potential treatment-related confounding. Moreover, most of these studies relied on laborious manual estimation of TILs. Although international efforts are underway to reduce variability and improve reproducibility in manual estimation of TILs, the outcome of such efforts in practice is still unclear. Computerised estimation of TILs can overcome many challenges in their assessment. Therefore, we evaluated the prognostic and predictive role of computationally characterised TILs in DCIS using the digitised pathology material from the UK/ANZ DCIS trial.

### Added value of this study

To the best of our knowledge, this is the first, largest, and most robust investigation of automated estimation of TILs in DCIS as a biomarker in a study nested within a randomised controlled trial (RCT) of DCIS. The prognostic and predictive value of the computationally evaluated TIL density was robustly demonstrated, thus providing a novel biomarker to personalise adjuvant treatment in DCIS.

### Implications of all the available evidence

Taken together with the other study nested in an RCT with a low risk of bias, our study provides supportive evidence for the prognostic role of TILs in DCIS. These findings—together with the significant interaction of computational TIL-based categories with radiotherapy—indicate that computational TIL categories can be used in clinical practice to personalise adjuvant radiotherapy decisions for patients with DCIS.
