# Supplementary material for: A prognostic and predictive computational pathology immune signature for ductal carcinoma in situ: retrospective results from a cohort within the UK/ANZ DCIS trial
Source: Lancet Digit Health. Author manuscript; Available in PMC 2025 Dec 30. (PMC12753184; doi:10.1016/S2589-7500(24)00116-X)
Supplement: Supplement [file NIHMS2125070-supplement-Supplement.pdf]

THE LANCET
Digital Health

Supplementary appendix

This appendix formed part of the original submission and has been peer reviewed. We post it as supplied by the authors.

Supplement to: Li H, Aggarwal A, Toro P, et al. A prognostic and predictive computational pathology immune signature for ductal carcinoma in situ: retrospective results from a cohort within the UK/ANZ DCIS trial. *Lancet Digit Health* 2024; published online July 9. https://doi.org/10.1016/S2589-7500(24)00116-X.

SUPPLEMENTARY MATERIAL:

CONTENTS:

Supplementary Figure S1

Full description of Nested case-control study design and details of additional statistical analyses

Supplementary table S1

Supplementary table S2

Supplementary table S3

Supplementary table S4

Supplementary Figure S2

Supplementary Section 1: calculation of Cpath TIL-categories in DCIS-associated micro-environment

Supplementary Figure S3

Supplementary Section 2: Reproducibility analysis

Supplementary Figure S4

Supplementary Figure S5

References

Supplementary Figure S1: CONSORT diagram summarizing the treatment allocation in the UK/ANZ DCIS trial, and distribution of patients in the biomarker study subset (BSS1) and inclusion in the nested case-control study.

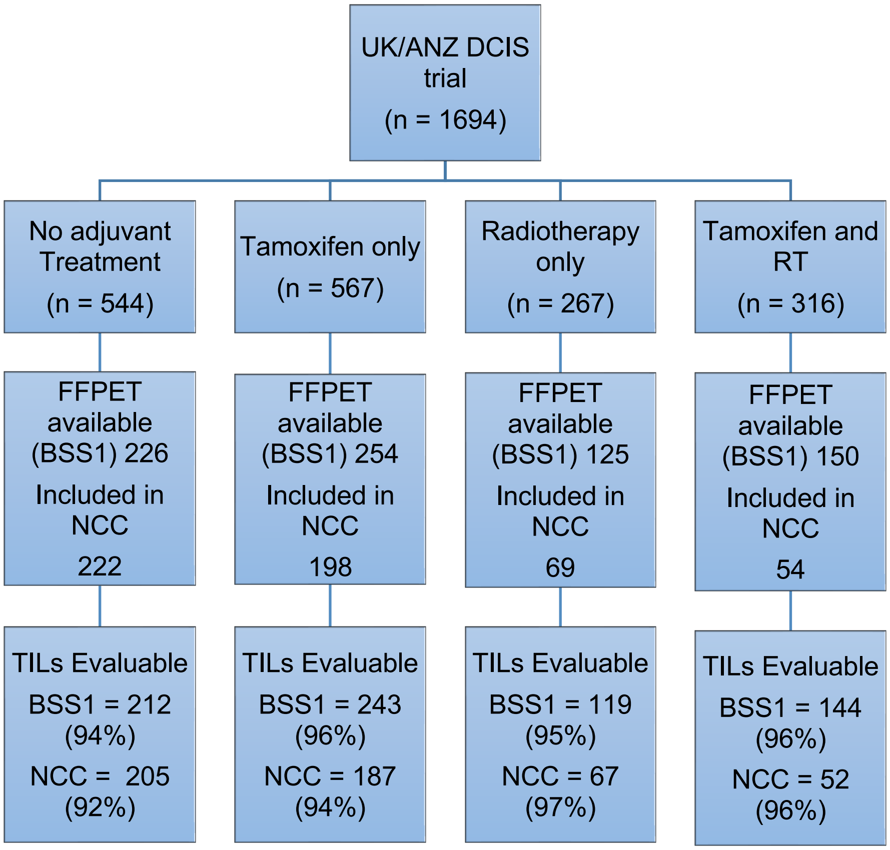

RT - radiotherapy; FFPET - Formalin-fixed paraffin embedded tissue; NCC - Nested Case-Control study; TILs – Tumour Infiltrating Lymphocytes.

Full description of Nested case-control study design

In order to minimize any residual treatment-related confounding, a nested case-control design was employed using a 1:2 case-control ratio. Overall, 566 controls (patients without recurrence) were available for matching to 189 cases (patients with any recurrence, either invasive or in situ), i.e. 3 potential controls for each case.

Similar to risk-set sampling (i.e. density sampling), the controls had to be followed up for at least the same length of time as the time-to-event in their matching case. Therefore, the pool of potential controls for an individual case, sampled at the time of event, included all event-free patients who had been followed up for at least the same length of time as the time-to-event in that particular case and this pool excluded all event-free patients with a shorter follow-up. From the available pool, controls were further matched to respective cases by age + 7 years and treatment allocation strata (12 strata). If more than 2 matching controls were available for a case, 2 of these were randomly selected. The first round of stringent matching done on the basis of 12 strata yielded 340 controls for 170 cases. This was then relaxed from matching by 12 strata to matching by 4 broad treatment groups (Supplementary figure 1), this resulted in 11 more cases being matched to 22 controls. The case-control study had a final sample size of 181 cases and 362 controls. The trial database is maintained using Oracle and a bespoke matching algorithm was developed for matching using programming language Oracle SQL Developer 4.x.

Full description of multivariate analysis

Besides univariate Cox analysis, multivariate Cox regression analysis was also performed to evaluate the independent prognostic significance of Cpath TIL signature by adjusting the impact of the clinicopathological and treatment variables on outcome. The covariates included age, HER2, completeness of excision, size (mm), cytonuclear grade, necrosis, adjuvant tamoxifen and adjuvant radiotherapy. To ascertain contribution of Cpath TIL signature, multivariate analyses with a baseline model of aforementioned covariates and with Cpath TIL signature added to this baseline model were performed. The likelihood ratio test $\chi 2$ values were compared.

Additional description of statistical analyses

Mann-Whitney test was employed to assess whether two independent groups come from populations with the same distribution or medians. While we used the Kruskal-Wallis test to determine if there is significant difference in data distribution between three or more independent groups. In addition, we employed Cochran-Armitage trend test to assess the association between a variable with two categories and an ordinal variable with three or more categories. The difference in the survival curves between two groups in KM curve plot was assessed by log-rank test with p value shown with the plot in the footnote.

Supplementary Table S1: Representativeness of subset where FFPET material was available.

Variable	Strata	BSS1	Remaining trial participants	χ^2	*p*
**Patients**	**N**	**755**	**939**		
**Age**	Mean (y) (SD)	57.5 (6.0)	57.2 (7.1)	0.46	0.50†
**Tumor size**	Mean (mm) (SD)	15.3 (n=673) (8.4)	14.1 (n=724) (10.6)	21.78	**<0.0001†**
**Cytonuclear Grade**	Low	38 (5.0%)	65 (6.9%)	11.50	**0.0007¶**
	Intermediate	114 (15.1%)	151 (16.1%)		
	High	518 (68.6%)	495 (52.7%)		
	No information	85 (11.3%)	228 (24.3%)		
**Necrosis**	Absent	51 (6.8%)	70 (7.5%)	5.67	**0.017**
	Present	617 (81.7%)	536 (57.1%)		
	No information	87 (11.5%)	333 (35.5%)		
**Excision**	Complete	474 (62.8%)	559 (59.5%)	0.597	0.44¶
	Uncertain	114 (15.1%)	96 (10.2%)		
	Incomplete	106 (14.0%)	123 (13.1%)		
	No information	61 (8.1%)	161 (17.2%)		
**RT**	Not given	480 (63.6%)	631 (67.2%)	2.43	0.12
	Given	275 (36.4%)	308 (32.8%)		
**Tamoxifen**	Not given	351 (46.5%)	460 (49.0%)	1.05	0.30
	Given	404 (53.5%)	479 (51.0%)		
**Recurrence**	No	566 (75.0%)	752 (80.1%)	8.99	**0.029**
	DCIS-IBE	94 (12.5%)	79 (8.4%)		
	I-IBE	59 (7.8%)	63 (6.7%)		
	Contralateral	34 (4.5%)	38 (4.0%)		
	No information	2 (0.3%)	7 (0.8%)		

¶: Cochran–Armitage trend test on 1 d.f.; †: Mann-Whitney test; Missing values, although provided in “No Information” row, are not treated as a separate category; BSS1 = biomarker study subset 1

Supplementary Table S2: Cpath TIL-categories (low, high) as a predictor of recurrence in the nested case-control study.

Case-definition	Cpath TIL-categories (low versus high)							
	Univariate				Multivariate			
	N	*Events*	mOR (95%CI)	*P*	N	*Events*	mOR (95%CI)	*P*
**IBE**	408	142	2.60 (1.66-4.09)	<0.0001	289	108	3.06 (1.56-6.03)	0.0012
**I-IBE**	160	54	2.37 (1.20-4.69)	0.013	113	42	4.29 (1.45-12.72)	0.0087
**DCIS-IBE**	242	86	2.58 (1.41-4.72)	0.0022	172	64	5.87 (1.64-20.99)	0.0065

$^{\phi}$recurrence type not known in 2 cases. Multivariate analyses are in a smaller number of samples due to non-availability clinicopathological data in some samples. mOR = Matched Odds Ratio.

Supplementary Table S3: Multivariate analysis of Cpath TIL-categories, clinicopathological and treatment variables with IBE as the case-definition in the nested case-control study.

Variable	Subgroup	*n*	Univariate		Multivariate	
			mOR (95%CI)	*p*	mOR (95%CI)	*p*
**Cpath TIL**	Low	126	1 (reference)	-	1 (reference)	-
	High	163	4.07 (2.23-7.43)	**<0.0001**	3.06 (1.56-6.03)	**0.0012**
**HER2**	Negative	177	1 (reference)	-	1 (reference)	-
	Positive (IHC3+)	112	2.46 (1.44-4.22)	**0.0010**	1.63 (0.84-3.13)	0.15
**ER**	Positive	167	1 (reference)	-	1 (reference)	-
	Negative	122	3.25 (1.90-5.56)	**<0.0001**	2.00 (1.04-3.83)	**0.037**
**Excision**	Complete	184	1 (reference)	-	1 (reference)	-
	Uncertain	55	1.10 (0.56-2.16)	0.77	1.05 (0.50-2.22)	0.90
	Incomplete	50	1.45 (0.76-2.77)	0.26	1.33 (0.63-2.82)	0.46
	Trend test			*0.27*		*0.54*
**Size (mm)**	mm (mean)	16.4	1.03 (1.00-1.05)	0.058	1.01 (0.98-1.04)	0.44
**Cytonuclear Grade**	Low	15	1 (reference)	-	1 (reference)	-
	Intermediate	36	0.99 (0.26-3.80)	0.99	0.46 (0.10-2.07)	0.31
	High	238	1.68 (0.53-5.33)	0.38	0.40 (0.10-1.66)	0.21
	Trend test			*0.14*		*0.28*
**Necrosis**	No	18	1 (reference)	-	1 (reference)	-
	Yes	271	2.52 (0.71-8.94)	0.15	1.48 (0.33-6.66)	0.61

Case-definition – IBE; n = 289; events = 108; univariate analyses restricted to the same sample size as available for multivariate analyses. mOR = Matched Odds Ratio. HER2-positive (IHC3+), HER2-negative (IHC 0, 1+, and 2+). ER by clonal method we have previously described.

Supplementary Table S4: 10-year event rates in Cpath TIL-categories by receipt of radiotherapy in BSS1.

Endpoint	Subgroup	10-year Event rate (95%CI)	
		Radiotherapy	
		No	Yes
**IBE**	Cpath TIL-low	17.4% (13.0%-23.1%)	7.9% (2.4%-4.3%)
	Cpath TIL-high	36.1% (30.1%-42.9%)	14.3% (3.1%-9.2%)
**I-IBE**	Cpath TIL-low	7.0% (4.2%-11.5%)	2.5% (1.4%-0.8%)
	Cpath TIL-high	14.8% (10.3%-20.9%)	7.6% (2.4%-4.0%)
**DCIS-IBE**	Cpath TIL-low	11.2% (7.7%-16.1%)	5.5% (2.0%-2.7%)
	Cpath TIL-high	24.0% (18.7%-30.4%)	7.3% (2.3%-3.8%)

Supplementary Figure S2: Illustration of overall workflow.

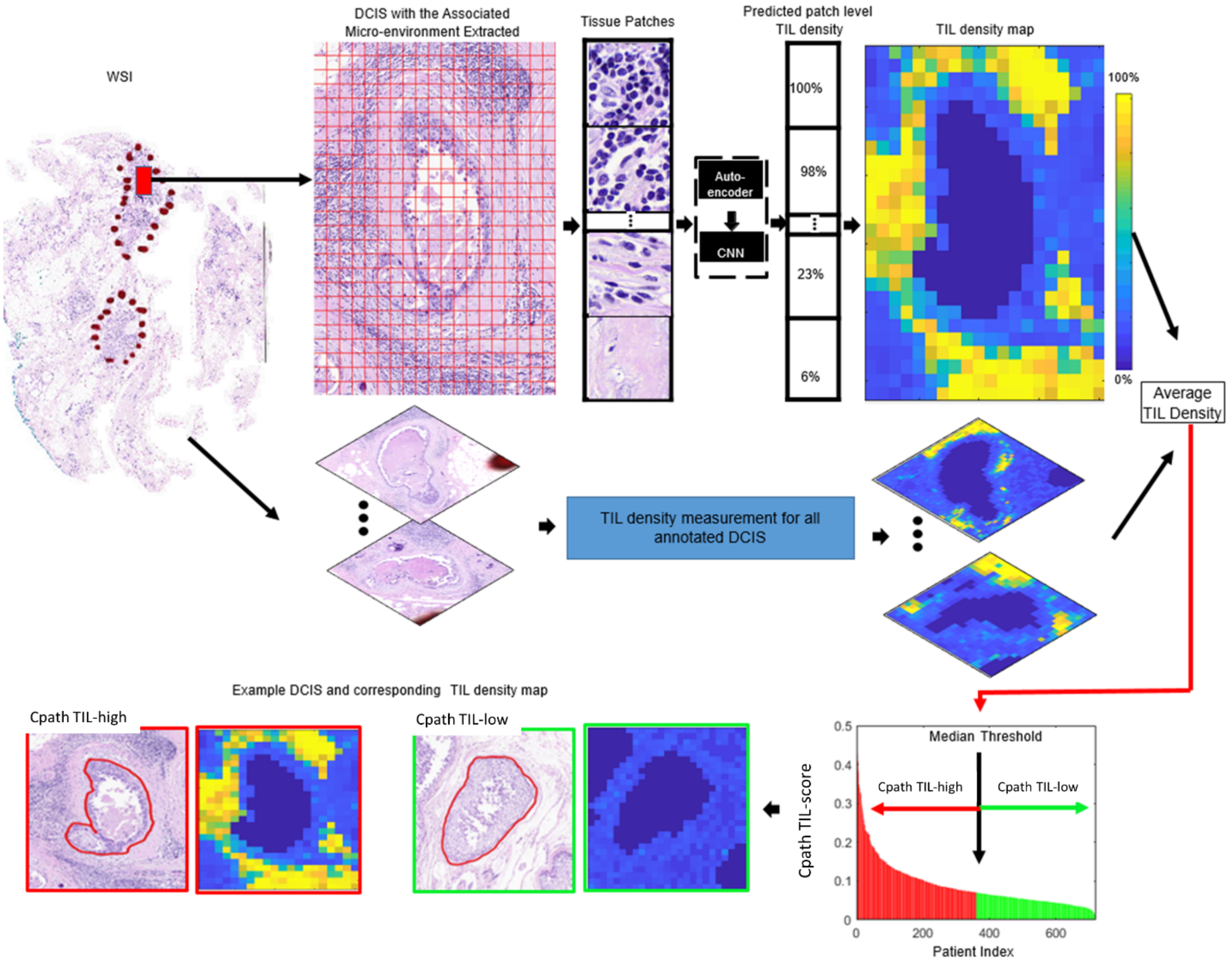

The DCIS-associated micro-environment for the manually annotated representative DCISs was extracted. The micro-environment was defined as the peripheral stroma region within a radius of 250 µm of computer-refined DCIS boundary. The micro-environment was split into an array of small image patches of size 50x50 µm^2. A CNN-based deep learning(1) model was employed to predict the TIL density (0%-100%) for each individual image patch and subsequently a TIL density map was generated for the DCIS-associated micro-environment. An approach combining a nuclei segmentation model(2) with image processing techniques was employed to segment the stromal areas. The biomarker named Cpath TIL-score was calculated as the average TIL density in the stromal region within DCIS-associated micro-environment across all the annotated DCISs within the WSI. Cpath TIL-categories were further generated by applying the median Cpath TIL-score to assign patients into a Cpath TIL-high/ Cpath TIL-low group.

Supplementary Section 1: calculation of Cpath TIL-categories in DCIS-associated micro-environment

Extraction of DCIS-associated micro-environment

Representative DCIS bearing area was annotated on the whole section images (WSI) by a pathologist. DCISs deemed too small for analysis by the pathologist were not manually annotated. The DCIS-associated micro-environment was defined as periphery region within a radius of ~250 μm of DCIS boundary. The radius of the micro-environment was visually regarded as reasonable for TIL study by an experienced pathologist.

Full description of stromal segmentation in DCIS-associated micro-environment

Stroma within the DCIS-associated micro-environment needs to be segmented for subsequent TIL analysis. The unannotated epithelium including small DCISs as indicated by the red arrows in Supplementary Figure S3 (1.b), and possibly non-DCIS epithelium such as atypical hyperplasia may be present in the micro-environment and may need to be further segmented and excluded from TIL density measurement. In addition, manual delineation of the DCISs by the pathologist may not be always highly precise due to the associated time burden as illustrated by the blue arrows in Supplementary Figure S3 (1.b). Accordingly, we developed an automatic procedure to accurately separate the stromal and epithelial regions, which in turn detected the un-annotated epithelium and refined the DCIS boundary delineation with the contours of the epithelium (non-stroma) mask as depicted in Supplementary Figure S3 (1.c). The stroma segmentation workflow was primarily based on dynamic searching for the optimal threshold of blue ratio (BR)(3) which in turn highlights Hematoxylin staining to separate the pixels of epithelium and stroma for any given individual slide level. The workflow is detailed in Supplementary S3 (2.a - 2.d). First, a pre-trained Hover-Net(2) was used to segment the nuclei (Supplementary Figure S3 (2.a)) in the DCIS and associated micro-environment. A morphologic image closing operation was subsequently performed on the Hover-Net generated nuclei mask

and the closed nuclei masks were utilized as a surrogate tissue epithelium mask (red color in Supplementary Figure S3 (2.b).). A staining deconvolution-based approach(4) was additionally used to detect the pen markings and associated white space, the goal being to mitigate the influence of these artifacts on the BR threshold selection. The tissue was then compartmentalized into surrogate epithelium (red), pen markings (blue), white space (yellow) and remaining area as surrogate stroma (green) as shown in Supplementary Figure S3 (2.b). Subsequently, as shown in Supplementary Figure S3 (2.c), an optimal threshold of BR was selected by maximally separating the distribution of pixel level BR for the surrogate epithelium and surrogate stroma masks respectively. The final epithelium and stroma masks were generated by binarizing the image based on the BR threshold (Supplementary Figure S3 (2.d)), followed by a set of morphological operations such as image closing and area opening to further optimize the segmentation for the DCIS versus the associated stroma [Supplementary Figure S3 (1.c)].

The performance of the approach for stroma segmentation was compared against a deep learning model for epithelium and stroma segmentation described in previous work(4), yielding a dice coefficient of 0.978 on a subset of DCIS slide images (N=661). The result suggests a high reliability and reproducibility of the stroma segmentation approach described in our study.

Details on the pre-trained CNN model for TIL classification in the stromal regions of the DCIS-associated micro-environment

The percentage of TILs present in each image patch was predicted by a pretrained CNN as described in Saltz's work(1). The rationale of utilizing the pre-trained CNN was that the model was trained on an extensive and diverse dataset comprising 5202 diagnostic slides spanning 13 TCGA tumor types, yielding an area under the receiver operating characteristic curve of 0.954 on a test set of TCGA studies. In addition, in our study, epithelium regions, which tend to present with more heterogeneous morphology and hence less reproducible for TIL

assessment(5), were excluded for TIL analysis by the CNN model. Focusing exclusively on the stromal area for TIL classification simplified the task, and despite CNN not being the most complex or cutting-edge model, it was deemed fit-for-purpose to meet the objectives of this study."

Model validation against ground-truth:

A semi-quantitative manual validation procedure was conducted to validate the performance of the pre-trained CNN model in our study. Specifically, five image patches with varying TIL density predicted by the CNN model were randomly cropped from the DCIS-associated stromal regions from a subset of patients(N=50) in our study. The five patches were arranged in a panel in the order of CNN-predicted low to high TIL-levels. A semi-quantitative assessment was conducted by a board-certified pathologist with 25-years of prior experience in breast cancer. One of four ranking categories: excellent, good, fair, and poor was assigned to the image patch panel by the pathologist, based on the accuracy of TIL level rankings within the panel.
The specific metrics for each category are as follows: Excellent: 5 patches were correctly ranked; Good: 4 patches were correctly ranked (ranking will be perfect after removing one patch); Fair: 3 patches were correctly ranked (ranking will be perfect after removing two patches); Poor: <=2 patches were correctly ranked. Based on the pathologist's assessment, 6% (N=3) were assigned as "Good" and 94% (N=47) were assigned as "Excellent""

Description of the blinded validation procedure

The average TIL density (Cpath TIL-score) in the DCIS micro-environment was finally calculated for each slide and dichotomized into binary groups (Cpath TIL-categories: Cpath TIL-low vs. Cpath TIL-high).

A blinded assessment was performed to validate the prognostic and predictive value of Cpath TIL-scores/ Cpath TIL-categories. Specifically, with the hypothesis that TIL density could be a prognostic and predictive biomarker for DCIS patients, a computational pipeline was

independently devised to quantify TIL density in DCIS-associated stroma by team 1. The WSIs of DCIS in the biomarker study subset nested within the UK/ANZ clinical trial were shared with team 1 by team 2. The slides were ingested into the pipeline to generate Cpath TIL-scores and Cpath TIL-categories, with all outcome and clinical variables withheld by team 2. The biomarker values were then returned to team 2 for blinded validation.

Supplementary Figure S3: workflow of stroma segmentation.

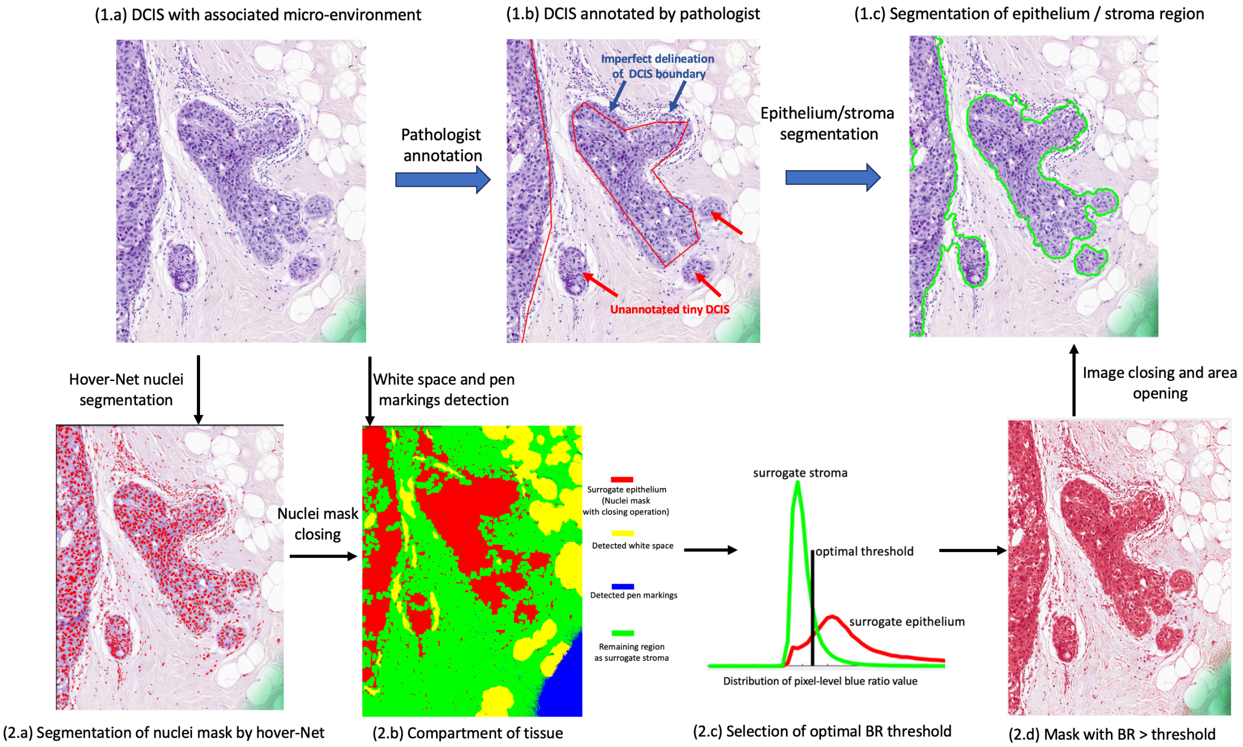

(1.a-1.c): Overall illustration of stromal segmentation in DCIS-associated micro-environment. Specifically, (2.a) a pre-trained Hover-Net was employed to segment the nuclei (red in 2.a). An Image closing operation was applied to the nuclei masks to generate the surrogate epithelium mask (red in 2.b). (2.b) Pen markings (blue) and white spaces (yellow) was additionally detected, and the remaining areas (green) were used as surrogate stroma masks. (2.c) An optimal BR threshold separating surrogate epithelium pixels and surrogate stroma pixels were also identified. (2.d): an intermediate epithelium mask (red) was acquired by applying the BR threshold, followed by a set of morphological operations to generate the final segmentation for stroma (epithelium contoured in green) (1.c).

Supplementary Section 2: Reproducibility analysis

The reproducibility of Cpath TIL-scores / Cpath TIL-categories was analyzed by investigating the correlation between TIL density estimated in this study and TIL density re-measured using alternative approaches. The same computational pipeline described in this study was independently implemented for all the patients for the biomarker measurement but utilizing alternative approaches for epithelium/stroma segmentation and TIL detection.

Specifically, a U-Net model described in previous work(6) was used for segmentation of the DCIS associated stroma. For TIL detection, a Hover-Net model(2) was first employed to segment the nuclei followed by a support vector machine(7) to classify the segmented nuclei as TIL versus non-TIL based on texture, shape and color related features.

The average and median value of measured Cpath TIL-scores was respectively 0.087 and 0.073, comparing with 0.088 and 0.071 for original Cpath TIL-scores. The Spearman correlation coefficient between original Cpath TIL-scores and the alternative segmentation Cpath TIL-scores was 0.95. The Cpath TIL-categories were further obtained by dichotomizing regenerated Cpath TIL-scores by applying median score as the threshold, with the Spearman correlation coefficient being 0.98 comparing against the original Cpath TIL-categories.

Alternative micro-environment radius:

To investigate the robustness of Cpath TIL-score/Cpath TIL-categories in relation to the selection of micro-environment radius, a radius of 200 μm was utilized to generate the Cpath TIL-score/Cpath TIL-categories. The Spearman correlation coefficient was 0.95 for Cpath TIL-score and 0.88 for Cpath TIL-categories between radius of 200 μm (median Cpath TIL-score=0.070) and radius of 250 μm (median Cpath TIL-score=0.071), with Cpath TIL-categories remaining the same for 94% patients.

Supplementary Figure S4: Ipsilateral recurrence risk by Cpath TIL-categories – Kaplan Meier survival plots of DCIS-IBE (A) and I-IBE (B) risk.

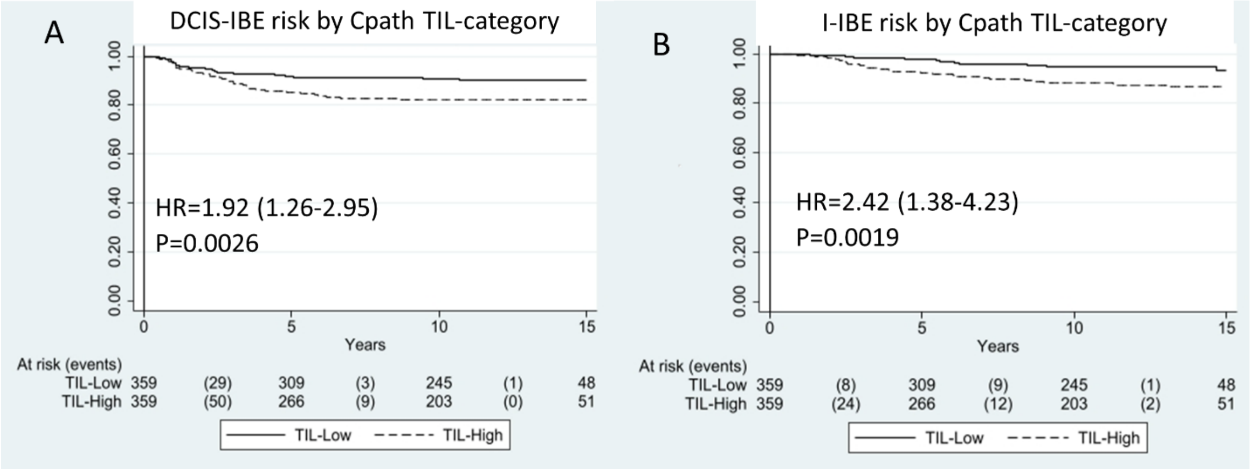

Log-rank p value = 0.0022 in (A) and p value <0.0001 in (B)

Supplementary Figure S5: Ipsilateral recurrence risk by Cpath TIL-categories in completely excised low or intermediate grade DCIS – Kaplan Meier survival plots of IBE risk.

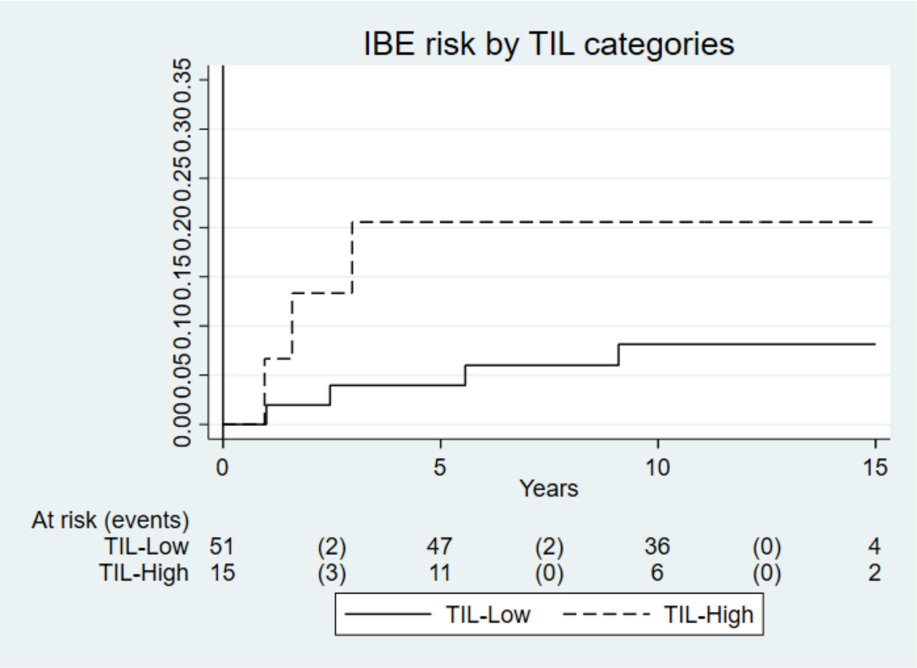

Note: Y-axis truncated at 0.35 for magnification. Log-rank p value = 0.14

References

1. Saltz J, Gupta R, Hou L, Kurc T, Singh P, Nguyen V, et al. Spatial Organization and Molecular Correlation of Tumor-Infiltrating Lymphocytes Using Deep Learning on Pathology Images. Cell Rep. 2018 Apr 3;23(1):181-193.e7.

2. Graham S, Vu QD, Raza SEA, Azam A, Tsang YW, Kwak JT, et al. Hover-Net: Simultaneous segmentation and classification of nuclei in multi-tissue histology images. Med Image Anal. 2019 Dec 1;58:101563.

3. Nuclear segmentation in H and E sections via multi-reference graph-cut (MRGC) – ScienceOpen [Internet]. [cited 2022 Jun 2]. Available from: https://www.scienceopen.com/document?vid=68795947-4500-4d05-8206-182c53a71246

4. Xu J, Xiang L, Wang G, Ganesan S, Feldman M, Shih NN, et al. Sparse Non-negative Matrix Factorization (SNMF) based color unmixing for breast histopathological image analysis. Comput Med Imaging Graph. 2015 Dec 1;46:20–9.

5. Thagaard J, Broeckx G, Page DB, Jahangir CA, Verbandt S, Kos Z, et al. Pitfalls in machine learning-based assessment of tumor-infiltrating lymphocytes in breast cancer: A report of the International Immuno-Oncology Biomarker Working Group on Breast Cancer. J Pathol. 2023 Aug;260(5):498–513.

6. Wu Y, Koyuncu CF, Toro P, Corredor G, Feng Q, Buzzy C, et al. A machine learning model for separating epithelial and stromal regions in oral cavity squamous cell carcinomas using H&E-stained histology images: A multi-center, retrospective study. Oral Oncol. 2022 Aug;131:105942.

7. Corredor G, Wang X, Lu C, Velcheti V, Romero E, Madabhushi A. A watershed and feature-based approach for automated detection of lymphocytes on lung cancer images. In: Medical Imaging 2018: Digital Pathology [Internet]. SPIE; 2018 [cited 2024 Feb 24]. p. 213–8. Available from: https://www.spiedigitallibrary.org/conference-proceedings-of-spie/10581/105810R/A-watershed-and-feature-based-approach-for-automated-detection-of/10.1117/12.2293147.full
